# Vitamin D Deficiency in HIV Infection: Not Only a Bone Disorder

**DOI:** 10.1155/2015/735615

**Published:** 2015-04-27

**Authors:** Pasquale Mansueto, Aurelio Seidita, Giustina Vitale, Sebastiano Gangemi, Chiara Iaria, Antonio Cascio

**Affiliations:** ^1^Department of Internal Medicine and Biomedicine, University of Palermo, 90100 Palermo, Italy; ^2^Department of Human Pathology, University of Messina, 98125 Messina, Italy; ^3^IFC CNR, Messina Unit, 98100 Messina, Italy; ^4^Infectious Diseases Unit, Papardo-Piemonte Hospital, 98125 Messina, Italy; ^5^AILMI-ONLUS Italian Association for the Control of Infectious Diseases, University of Messina, 98125 Messina, Italy

## Abstract

Hypovitaminosis D is a worldwide disorder, with a high prevalence in the general population of both Western and developing countries. In HIV patients, several studies have linked vitamin D status with bone disease, neurocognitive impairment, depression, cardiovascular disease, high blood pressure, metabolic syndrome, type 2 diabetes mellitus, infections, autoimmune diseases like type 1 diabetes mellitus, and cancer. In this review, we focus on the most recent epidemiological and experimental data dealing with the relationship between vitamin D deficiency and HIV infection. We analysed the extent of the problem, pathogenic mechanisms, clinical implications, and potential benefits of vitamin D supplementation in HIV-infected subjects.

## 1. Introduction

Human immunodeficiency virus type-1 (HIV) is a global health problem that has infected 60 million people and caused 25 million deaths worldwide. To date, it has been estimated that more than 33 million people, including 2 million children, live infected by HIV. However, even if the problem is far from a definitive solution, highly active antiretroviral therapy (HAART) has profoundly changed the natural history of HIV infection dramatically reducing AIDS- (acquired immune deficiency syndrome-) related morbidity and mortality [1]. Nevertheless, at least until now, HAART cannot eradicate HIV [2, 3]. Increased life expectancy exposes HIV-infected subjects both to chronic adverse drug reactions and to age-related morbidities, including neurocognitive disorders, cardiovascular and metabolic disease, renal and bone diseases (i.e., osteopenia/osteoporosis), and cancer [4–6]. Many of these appear to occur earlier in HIV patients compared to the general population. Key factors explaining premature age-associated non-AIDS-related events in patients receiving HAART are chronic inflammation and immune activation [7, 8]: plasma levels of several inflammatory and coagulopathy biomarkers, such as interleukin-6 (IL-6), highly sensitive C-reactive protein (hsCRP), and D-dimer are higher and correlate with outcome in HIV infection [9, 10].

Considering the potential role of vitamin D in many of these chronic illnesses, the scientific community focuses attention on the possible impact of its deficiency on the HIV-infected population. In this review, we first briefly describe vitamin D metabolism and its biological functions; then, we focus on the most recent epidemiological and experimental data dealing with the relationship between vitamin D deficiency and HIV infection. We analyse the extent of the problem, pathogenic mechanisms, clinical implications, and the potential benefits of vitamin D supplementation among HIV-infected subjects. We researched the PubMed database for the period from 1980 through January 31, 2015, using the keywords “HIV,” “vitamin D,” “neurocognitive disorders,” “cardiovascular disease,” “metabolic disease” (i.e., diabetes and metabolic syndrome), “renal disease,” and “cancer.” Articles presenting original data as well as reviews were included in our analysis.

## 2. Prevalence of Hypovitaminosis D in HIV-Infected Subjects

Hypovitaminosis D is a worldwide disorder, with a high prevalence in the general population of both Western and developing countries. It has been estimated that more than 1 billion people suffer from either 25(OH)D (25-hydroxyvitamin D) deficiency or insufficiency. According to the results of the National Health and Nutrition Examination Survey (NHANES), 25(OH)D deficiency and insufficiency are at 79% among adults [11]. Thus, like the general population, it is not surprising to find high rates of hypovitaminosis D among HIV-infected subjects. The overall estimated prevalence in people living with HIV and 25(OH)D deficiency is high, ranging from 70.3 to 83.7% (Table 1).

Eckard et al. conducted an investigation on hypovitaminosis D and the possible variables associated with this pathological framework in HIV-infected pregnant women and their infants compared to healthy controls. It was found that 25(OH)D concentrations in serum cord blood were <30 ng/mL in 100% of subjects from both groups. The only variables associated with higher serum 25(OH)D concentrations were white race and non-Hispanic ethnicity [12]. These data agreed with previous observations asserting that vitamin D deficiency not only contributes to HIV disease progression and mortality in HIV-infected pregnant women, but also increases the overall risk of mother-to-child transmission by 46% [13] and the risk of death in newborns by 61% during follow-up [13]. While most infants born to HIV-infected mothers in the US will not acquire HIV infection, in utero ART (antiretroviral therapy) exposure may increase their cancer risk later in life. Thus, maternal and therefore infant 25(OH)D deficiency should not be disregarded [14].

## 3. Risk Factors for Vitamin D Deficiency in HIV-Infected Subjects

In the setting of HIV infection, 25(OH)D deficiency may be affected by both HIV-related and -independent risk factors; however, it is often challenging to differentiate the direct impact of HIV infection from the effect of traditional risk factors which may be normal or overexpressed in HIV-positive cohorts.

### 3.1. HIV-Related Risk Factor

The relationship between 25(OH)D levels, viral load, and CD4+ T-cell count is not clear cut. Some studies described a positive correlation [15, 16], some others failed to demonstrate a significant association [17, 18], and, finally, others did not find that vitamin D (in any possible formula) supplementation can increase CD4+ count [19, 20]. Different mechanisms have been hypothesized to explain the association between 25(OH)D deficiency and higher severity of HIV disease. First, 25(OH)D deficiency may be a contributory causal agent of the HIV infection itself. Second, chronic inflammation due to HIV infection and subsequent TNF-*α* overproduction may be responsible for renal 1*α*-hydroxylase impairment, reducing the PTH (parathyroid hormone) stimulatory effect on the production of the hormonally active 1,25(OH)2D (1,25-dihydroxyvitamin D). Third, infectious complications as a result of poor immunity require hospital care, which significantly reduces the duration of sun exposure for patients. Lastly, both infectious complications and hospitalization may lead to malnutrition and reduced oral intake of the few foods that contain vitamin D [21, 22].

### 3.2. HIV-Independent Risk Factors

Several traditional hypovitaminosis D risk factors, such as female sex, increasing age, reduced exposure to sunlight, winter season, dark skin pigmentation, non-Caucasian race (i.e., African American ethnicity), greater body mass index (BMI), low vitamin D dietary intake, gastrointestinal absorption disorders, liver and renal diseases, multiple cardiovascular disease risk factors, including diabetes mellitus, and current alcohol consumption, are similar in both HIV-positive and HIV-negative cohorts [23, 24]. An exception is represented by intravenous drug use, which has not been extensively studied in the general population [25]. Injection drug users (IDUs) often have poor nutritional status and limited/delayed access to healthcare. In addition, intravenous drug use increases the risk for a host of acute and chronic infectious and cardiopulmonary conditions. As a result, this patient population suffers a disproportionate burden of 25(OH)D deficiency, compared to other urban dwelling adults [26, 27]. In 2014, Lambert et al. evaluated the relationship between intravenous drug use, 25(OH)D deficiency, and HIV infection, analysing 950 individuals (29% of them were HIV-infected). The study found that 74% of subjects were 25(OH)D deficient (68% in HIV-infected versus 76% in HIV-uninfected, *P* = 0.01); significantly, higher odds of 25(OH)D deficiency were observed in black race, late winter/early spring season, lack of multivitamin use, and hypoalbuminemia (the latter as an expression of poor nutritional state). Notably, HIV- and HCV-infected IDUs were less likely to be 25(OH)D deficient, evoking questions regarding the role of free vitamin D measurement (not influenced by albuminemia) in these unique populations [25].

## 4. Vitamin D Status and HAART

Recently several* in vitro* and* in vivo* studies focused on the impact of antiretroviral drugs on vitamin D metabolism. Both protease inhibitors (PIs) and nonnucleoside reverse transcriptase inhibitors (NNRTIs) have been associated with the impairment of vitamin D metabolic pathways [28–30].

PIs, especially darunavir and ritonavir, seem to interfere with vitamin D metabolism by inhibition of vitamin D 1*α*- and 25*α*-hydroxylation both in hepatocyte and in monocyte cultures: reduction of 25(OH)D conversion to its active metabolite may potentially explain the reports of increased 25(OH)D levels in subjects with low 1,25(OH)2D. Regarding NNRTIs, there is an increasing amount of experimental data associating efavirenz (EFV). Unlike what was just reported for PIs, EFV seems to increase 25(OH)D catabolism and production of inactive metabolites, through the interaction with cytochrome P450 enzymes, some of which may affect vitamin D metabolism (i.e., induction of CYP24A1 [31, 32] and reduced transcription of CYP2R1), similar to the effects of antiepileptic drugs [33]. This hypothesis has been supported by several* in vivo* studies, which described an association between NNRTIs, especially EFV and nevirapine (NVP) use and low 25(OH)D levels (Table 2).

The weakness of most reported studies is the cross-sectional design, so that causal relationships cannot be inferred. These data suggest the need for large prospective studies, properly designed to evaluate the specific effects and clinical impact of antiretroviral drugs on vitamin D status.

## 5. Association between HIV, Hypovitaminosis D, and Cardiovascular Disease

Several studies have described the association between HIV and increased risk of CVD (cardiovascular disease) [34, 35]. HIV infection itself is considered an independent risk factor for atherosclerosis: the prevalence of carotid intima-media thickness (cIMT), atherosclerosis, and myocardial infarction is higher among HIV-positive subjects, occurring earlier compared to uninfected individuals [36, 37]. In these patients atherogenesis is enhanced by several factors: HIV-induced chronic inflammation and immune activation (demonstrated by increased levels of proinflammatory cytokines and endothelial activation markers), excess of traditional risk factors (e.g., 2 to 3 times higher prevalence of smoking), and antiretroviral drug-related dyslipidemia, hyperglycemia, central obesity, and lipodystrophy (especially with PIs) [38–40]. To make this framework even more complex, 1,25(OH)2D deficiency has been linked to CVD in the general population [41, 42]. Vitamin D influences cardiovascular health by suppressing the renin-angiotensin system and stimulating cellular proliferation and differentiation via 1,25(OH)2D binding to vitamin D receptors in the heart, the endothelium, and the vascular smooth muscle [43, 44].

### 5.1. cIMT, Brachial Artery Flow-Mediated Dilation, Coronary Artery Calcium (or Calcification), and Coronary Artery Stenosis

Considering the high prevalence of both hypovitaminosis D and CVD in patients with HIV, the evidence of a relationship between low 25(OH)D and silent and symptomatic atherosclerosis is not surprising. Even though in the general population asymptomatic CVD, as demonstrated by cIMT, brachial artery flow-mediated dilation (FMD, an early marker of endothelial dysfunction), and CAC (coronary artery calcification), has been strongly linked to the occurrence of cardiovascular events and has also been independently associated with 25(OH)D deficiency, only a few studies are available in HIV-infected populations; moreover, none of these studies shows if 25(OH)D repletion might affect cardiovascular outcomes. The clinical characteristics of the populations, the study designs, and the variables included in the analysis of results could explain the differences among the studies [45–47] (Tables 3 and 4).

### 5.2. Other Risk Factors for CVD in HIV-Infected Subjects

Other traditional risk factors for CVD, such as insulin resistance and diabetes mellitus, are frequently seen in HIV-positive individuals [48], and, as in the general population [49], an association between vitamin D status and type 2 diabetes, but not with insulin resistance, has been described [50, 51]. A recent Italian cross-sectional study of 1811 HIV-infected persons, enrolled in the prospective Modena (Italy) HIV Metabolic Clinic Cohort, reported lower 25(OH)D levels in subjects with Type 2 diabetes, compared to those without diabetes (*P* < 0.001), although 25(OH)D deficiency was highly prevalent in both groups. In addition, although 25(OH)D deficiency was independently associated with diabetes (OR 1.85; CI 1.03–3.32, *P* = 0.038), the association with metabolic syndrome was not significant after adjusting for vitamin D supplementation, sex, age, and BMI (adjusted OR 1.32; 95% CI 1.00–1.75; *P* = 0.053) [50]. In the setting of HIV few data are available and the effects of vitamin D(3) supplementation on insulin sensitivity need to be evaluated with large, prospective studies. However, surprising results were provided by a small prospective study conducted by van den Bout-van den Beukel et al., which showed that cholecalciferol supplementation (2,000 IU/day for 14 weeks, 1,000 IU/day until 48 weeks) led to increased HOMA measured insulin resistance, after 24 weeks, whereas no differences were seen after 48 weeks [52]. It remains to be clarified whether the results are dose- or time-dependent, but this report further suggests the importance of clinical trials extensively evaluating the pros and cons of supplementing HIV-infected individuals with cholecalciferol [52].

## 6. Association between HIV, Hypovitaminosis D, and HIV Disease Progression

Preclinical experiments have demonstrated that treatment of peripheral blood mononuclear cells with 1,25(OH)2D decreased the cell susceptibility to HIV infection by inhibiting viral entry, modulating expression of CD4+ cell surface antigen, damping viral p24 production, and limiting monocyte proliferation [53, 54]. Thereafter, several observational studies have shown a significant association between higher levels of 25(OH)D and rates of immune recovery [15, 55, 56]. Along these lines, some authors investigated the association between vitamin D and clinical outcomes. Baseline 25(OH)D levels lower than 32 ng/mL were independently associated with progression to more advanced HIV stage among 884 HIV-infected pregnant women in Tanzania, who were followed for a median of 70 months. The women with 25(OH)D in the highest quintile had a 42% lower risk of all-cause mortality than the women in the lowest quintile [13]. The same authors demonstrated that 25(OH)D deficiency was also associated with low BMI, oral thrush, acute upper respiratory infections, and severe anemia [57]. However, other studies failed to demonstrate an association between 25(OH)D level and clinical outcome, as in the above mentioned study by Sherwood et al. [58].

## 7. Association between HIV, Hypovitaminosis D, and Hepatitis C

HCV (hepatitis C virus) infection occurs at a significantly higher rate in HIV-infected persons compared to the general population, and this is especially problematic for resource-limited settings, where HCV treatment is generally not easily available [59]. HIV has a negative impact on the natural history of HCV, and, compared to HCV monoinfected patients, HIV/HCV coinfected patients have a more rapid progression from chronic active hepatitis to liver-cirrhosis, end-stage liver disease, liver cancer, and death, as well as lower response rate to traditional HCV treatment [60, 61]. Male sex, insulin resistance, acquiring HCV at an older age, heavy alcohol consumption, HCV genotype 3, and low CD4+ cell count are the factors contributing to the rapid development of liver fibrosis/cirrhosis among HIV/HCV coinfected patients [62, 63]. Other studies of HCV monoinfected patients have shown an independent association of 25(OH)D deficiency with severe liver fibrosis and treatment failure [64].

1,25(OH)2D effects on the immune system and inflammatory response have been shown to directly inhibit the proliferation and profibrotic effect of hepatic stellate cells [65]. Not surprisingly, liver fibrosis is associated with low serum levels of 25(OH)D during both HBV- and HCV-related chronic hepatitis, in both HIV-coinfected and not-coinfected patients [66]. However, low levels of 25(OH)D have been found in HBV or HCV carriers with minimal or absent liver fibrosis compared to healthy subjects [67].

On the other hand, in HIV-HCV coinfected patients, studies investigating the association between HCV sustained virologic response and vitamin D level have reported varying results, with some studies demonstrating an association [68], whereas other studies do not [69]. Mandorf et al. demonstrated that serum levels of 25(OH)D may predict the response to anti-HCV therapy. Suspicion of such a connection is strengthened by the evidence that cholecalciferol supplementation improves early and sustained virological response (94% versus 48% in controls and 86% versus 42% in controls, resp.) in HCV genotype 1 patients treated with Peg-IFN/ribavirin [70, 71]. The exact mechanism of its antiviral (anti-HCV) effect is unknown, although it was recently shown to amplify the innate antiviral immune response upregulating IFN-*β* and the MxA (an IFN-induced human protein) gene expression and dampening interferon gamma-induced protein 10 (IP-10) expression [72].

## 8. Association between HIV, Hypovitaminosis D, and Tuberculosis

According to the World Health Organization approximately 2 billion people are exposed to* M. tuberculosis*, 8 million people per year are infected, and 2 million people die as a clinical outcome [73]. HIV is the strongest factor in the development of active TB (tuberculosis), and its spread has fuelled the resurgence of the TB epidemic. It has been proposed that in HIV infection* M. tuberculosis* escapes the local immune response within the granulomas, decreasing their containing ability and then leading to increased mycobacterial replication, dissemination, and clinical disease [74]. The rise in CD4+ count and improved immune function after HAART initiation partially restore pathogen specific immunity. In the general population, 25(OH)D levels lower than 4 ng/mL were shown to cause a 3-fold probability of having active TB [75], with higher risk of developing MDR* M. tuberculosis* infection [76]. A cross-sectional study of 174 HIV-infected and 196 HIV-uninfected individuals in Cape Town, South Africa, showed that 25(OH)D deficiency is independently associated with active TB and this association is greater in HIV-infected subjects [77]. A prospective Tanzanian cohort study enrolled 1103 HIV-infected persons initiating HAART in a randomized controlled trial (RCT) of vitamin D-free multivitamin supplementation. Baseline 25(OH)D levels lower than 20 ng/mL, but not 25(OH)D insufficiency, were associated with higher incident smear-positive TB, after a median follow-up of 20.6 months, wasting, and >10% weight loss but not with risk of malaria, pneumonia, or anaemia. Mortality hazard ratio was 2.0 for those with levels below 20 ng/mL versus those with levels above 30 ng/mL over 24 months. Reverse causality (i.e., that vitamin D deficiency occurred as a result of TB) was ruled out in this study by the exclusion of patients who developed TB within 1 month of enrolment. This finding is significant, since TB itself might contribute to vitamin D deficiency by reducing a patient's sun exposure or increasing consumption of 25(OH)D by activated macrophages [78]. Recently a systematic review was conducted to analyse studies published from 1980 to 2006 with data on serum 25(OH)D in pulmonary TB patients and controls. Five out of seven case-control studies, with a total of 531 participants, reported lower serum 25(OH)D in cases compared to controls. Several weaknesses were found: the sample sizes were small, ranging between 30 and 145 participants; some studies did not use culture for diagnosing TB; some studies included extrapulmonary TB; selection of controls was not optimal [79].

## 9. Association between HIV, Hypovitaminosis D, Chronic Inflammation, and Malignancy

### 9.1. Chronic Inflammation

HIV infection is associated with chronic inflammation (i.e., elevated TNF, IL-6, and CRP) and immune system activation (i.e., increased soluble CD14 and CXCL10), even after achieving full virologic suppression and immune recovery with the use of HAART [80, 81]. In this population, elevation of inflammation markers has been shown to be independent predictors of neurocognitive impairment, frailty, cardiovascular events, diabetes and metabolic syndrome, low BMD, malignancies, and all-cause mortality [82–85]. The same outcomes, including all-cause mortality, were also associated with chronic inflammation in the general population [86]. Thus, there seems to be a considerable overlap in the outcomes associated with 25(OH)D deficiency and chronic inflammation, in both the HIV-infected and HIV-uninfected populations.

### 9.2. Malignancy

Association of vitamin D deficiency with risk of cancer in the HIV-infected population remains to be determined. However, it has already been shown in the general population, including breast cancer (4-fold risk) [87], colon cancer (2-fold risk) [88], ovarian cancer (4-fold risk) [89], and prostate cancer (3-fold risk) [90]. However, to date, there is only one study that tried to correlate 25(OH)D deficiency, HIV, and cancer. Erlandson et al. enrolled 90 HIV-infected patients with AIDS-associated Kaposi's sarcoma (KS) from Zimbabwe, in a prospective pilot study investigating the effect of antiretroviral therapy on the natural history of this neoplasm. The authors demonstrated that 25(OH)D insufficiency was common and HIV-1 RNA was significantly higher in those with insufficient 25(OH)D; in contrast, tumor response, survival, and KS-associated immune reconstitution inflammatory syndrome (defined as any progression of KS occurring ≤12 weeks after initiation of HAART) were generally associated with an increased CD4+ lymphocyte count of at least 50 cells/mL above the baseline value, before or at the time of documented KS progression, and were not associated with 25(OH)D status [91].

## 10. Management of Hypovitaminosis D in HIV-Positive Individuals

### 10.1. Screening

The main arguments in favor of routine screening of vitamin D in HIV-infected patients include the potential optimization of skeletal, metabolic, and immunologic parameters with vitamin D supplementation. The arguments against routine screening include assay variability and costs, lack of a clear target range, absence of proven supplementation benefits, apart from the benefits connected with osteoporosis as in the general elderly population, limited randomized clinical trial data in HIV-infected patients, inability to distinguish the effects of vitamin D and calcium supplementation on bone, potential harm from some supplementation approaches, and increased pill regimen (possible reduction of patient compliance).

The European AIDS Clinical Society most recent guidelines suggest vitamin D status evaluation in patients with a history of low BMD or fracture, those with high risk of fracture, or those with other vitamin D deficiency associated factors (e.g., persons receiving some antiretroviral drugs, including EFV). Vitamin D replacement is recommended when 25(OH)D is lower than 10 ng/mL; for values ranging between 10 and 20 ng/mL, supplementation is recommended only for patients with osteomalacia, osteoporosis, or increased PTH [92]. McComsey et al. developed recommendations for bone disease in HIV infection, addressing vitamin D deficiency as well. They recommend 50,000 IU of cholecalciferol weekly for 8 to 12 weeks and then monthly thereafter or 2,000 IU daily for 12 weeks and then 1,000 to 2,000 IU daily thereafter. 25(OH)D levels after replacement should be measured. They recommend supplementation to achieve 25(OH)D greater than 32 ng/mL [93].

### 10.2. Supplementation

In the general population, recommendations regarding vitamin D supplementation are mostly derived from studies on bone health. Several large RCTs found beneficial effects of vitamin D* plus* calcium on BMD and fracture risk [94]. Meta-analyses showed that vitamin D (cholecalciferol)* plus* calcium association is superior to the use of a single drug in fracture prevention [94]. Unfortunately, the evidence for vitamin D use in clinical outcomes beyond skeletal health (i.e., on falls, CVD, diabetes, metabolic syndrome, immune response, and cancer) is inconsistent with and insufficient to base general recommendations.

#### 10.2.1. Vitamin D Supplementation Dosage

In the general population, current recommended vitamin D oral supplementation is 800–1,000 IU cholecalciferol/day, plus calcium 1000 mg to 1200 mg daily. Serum 25(OH)D levels generally increase by approximately 1 ng/mL for every 100 IU of cholecalciferol intake. Few data from small cohorts are available on the efficacy of cholecalciferol repletion in HIV-infected subjects [95]. However, in this subgroup, it seems that a daily intake of at least 1,000–2,000 IU/day may be needed to overcome vitamin D deficiency [29].

#### 10.2.2. Safety of Vitamin D Supplementation

Groleau et al. demonstrated that supplementation with high vitamin D(3) doses and the concomitant increased serum 25(OH)D level did not correlate with increased whole blood lead concentration in HIV-infected children and young adults. Vice versa, the more robust increase in serum 25(OH)D after 12 weeks of vitamin D(3) supplementation for participants enrolled during winter and spring was accompanied by a decrease in whole blood lead concentration [96]. Animal studies show an inverse relationship between calcium intake and lead levels. This inverse relationship was also found in pregnant women, and calcium supplementation during pregnancy was associated with reductions in blood lead. Overall, the above data provide safety information when considering higher dose vitamin D intervention [97].

#### 10.2.3. Extraskeletal Effects of Vitamin D Supplementation

There are only a few studies investigating the effect of cholecalciferol supplementation on other cardiovascular, metabolic, and immunological outcomes in the HIV-infected population. In an RCT involving 45 subjects with 25(OH)D lower than 20 ng/mL, 12-week supplementation with daily oral cholecalciferol 4,000 IU produced an increase of approximately 5 ng/mL in 25(OH)D level compared to placebo but did not result in a statistically significant change in brachial artery FMD. Moreover, in the study group, insulin resistance increased from baseline but it was not statistically different from the placebo arm; similarly, baseline inflammatory and coagulation markers (i.e., CRP, IL-6, sTNFR-1, ICAM, vascular cell adhesion molecule (VCAM), D-dimer, and fibrinogen) did not significantly change between the groups. These results could partly be attributed to the modest increase in 25(OH)D (5 ng/mL) in subjects receiving cholecalciferol supplementation [98]. In an RCT involving 52 mostly virologically suppressed vertically infected youths aged 8 to 26 years with 25(OH)D lower than 30 ng/mL, Giacomet et al. showed that 12-month supplementation with cholecalciferol 100,000 IU every 3 months resulted in reduction of anti-inflammatory T-cell phenotype (i.e., decrease in T_H_17 : T_reg_ ratio) at 3 months. This effect was no longer seen at 12 months. No significant change in baseline CD4+ cell count was observed between the treatment and placebo arms [99].

## Figures and Tables

**Table 1:** Prevalence of hypovitaminosis D in HIV-infected subjects reported by nation.

Authors (year), journal	Nation	Patients	Results	Comments
Dao et al. (2011) [23], Clinical Infectious Diseases	US	672 HIV-positive patients versus US general population.	70.3% patients had 25(OH)D levels below 30 ng/mL versus 79.1% of HIV-negative US adults.	Vitamin D deficiency was not different between the two groups and no relationship could be found with duration since HIV diagnosis and vitamin D deficiency.

Adeyemi, Agniel et al. (2011), Journal of Acquired Immune Deficiency Syndromes	US	1268 HIV-positive versus 510 HIV-negative women.	60% patients had 25(OH)D levels below 20 ng/mL versus 72% of controls.	Vitamin D deficiency was found in total 63% of women with the highest rates in African American women. No other predictive factors of hypovitaminosis were found in multivariate analysis.

Eckard, Judd et al. (2012), Antiviral Therapy	US	200 HIV-infected and 50 HIV-uninfected youth Americans.	77% of HIV-positive and 74% of controls had 25(OH)D <20 ng/mL.	No difference in 25(OH)D was proved between groups. However, with a 77% and 96% prevalence of vitamin D deficiency and insufficiency, nearly all HIV-infected youth suffered from these conditions.

Poowuttikul, Thomas et al. (2014), Journal of the International Association of Providers of AIDS Care	US	160 HIV-infected youth.	5% had normal 25(OH)D levels; 23.1% had 25(OH)D levels between 21 and 35 ng/mL; 71.9% had 25(OH)D level ≤20 ng/mL.	Severe vitamin D deficiency (25(OH)D ≤10 ng/mL) was related to lower CD4 counts and CD4% but not to HIV plasma RNA. CD4 counts/CD4% did not increase under vitamin D supplementation.

Crutchley, Gathe et al. (2012), AIDS Research and Human Retroviruses	US	200 HIV-infected patients.	64% had 25(OH)D <20 ng/mL and 20.5% had 25(OH)D <10 ng/mL.	Multivariate analysis showed a significant correlation between low 25(OH)D levels, African-American race, and low daily vitamin D supplemental intake.

Stein, Yin et al. (2011), Osteoporosis International	US	89 HIV-positive and 95 HIV-negative postmenopausal women (33% Afro-Americans and 67% Hispanic).	74% of HIV-positive versus 78% of HIV-negative women had 25(OH)D <30 ng/mL.	25(OH)D was significantly lower in Afro-American subjects and higher in subjects who used both calcium and multivitamins. 25(OH)D level was directly associated with current CD4 count (*P* < 0.01). No association was observed between 1,25(OH)(2)D and CD4 count or between serum 25(OH)D, 1,25(OH)(2)D, and type of cART.

Kwan, Eckhardt et al. (2012), AIDS Research and Human Retroviruses	US	463 HIV-infected patients.	24% 25(OH)D <30 ng/mL (insufficiency) 59% 25(OH)D <20 ng/mL (deficiency).	In this population, hyperparathyroidism prevalence was 30% in patients with vitamin D deficiency, 23% in those with insufficiency, and 12% in those with sufficient vitamin D levels.

French, Adeyemi et al. (2011), J Womens Health (Larchmt)	US	602 nonpregnant (480 HIV-infected and 122 uninfected) subjects.	24.4% 25(OH)D <30 ng/mL (insufficiency) 59.4% 25(OH)D <20 ng/mL (deficiency).	Only race was significantly associated with vitamin D deficiency, with no differences in HIV status.

Yin, Lu et al. (2010), Journal of Acquired Immune Deficiency Syndromes	US	100 HIV-positive and 68 HIV-negative premenopausal women.	91% of HIV-positive and 91% of HIV-negative had 25(OH)D levels <32 ng/mL; 69% of HIV-positive and 60% of HIV-negative had 25(OH)D levels <20 ng/mL; 30% of HIV-positive and 24% of HIV−negative had 25(OH)D <10 ng/mL.	In premenopausal HIV+ women, bone mineral density was lower than comparable HIV-women. Vitamin D level was not associated with differences in HIV status.

Rodriguez, Daniels et al. (2009), AIDS Research and Human Retroviruses	US	57 HIV-positive patients.	36.8% patients had 25(OH)D <20 ng/mL. 10.5% patients had 25(OH)D <10 ng/mL.	Lower vitamin D intake was significantly associated with severe 25(OH)D deficiency. Lactose intolerance tended to be associated with severe 25 (OH)D deficiency.

Wasserman and Rubin (2010) [17], AIDS Patient Care STDS	US	19 HIV-positive patients under NNRTI versus 37 HIV-positive patients under PI.	73.7 NNRTI recipients had 25(OH)D <50 nmol/L. 29.7 (11/37) PI recipients had 25(OH)D <50 nmol/L.	Vitamin D deficiency was not correlated to stable viral suppression. HAART receipt and tobacco use were associated with lower vitamin D levels and greater risk of deficiency and severe deficiency, respectively.

Viard et al. (2011) [21], AIDS	31 European countries, Israel, and Argentina	1985 HIV-positive among EuroSIDA study group.	23.7% had 25(OH)D <10 ng/mL. 65.3% had 25(OH)D between 10 and 30 ng/mL. 11% had 25(OH)D >30 ng/mL.	As in the general population, season (winter), age (older), and race (black) affected 25(OH)D levels (reduction). Hypovitaminosis D was independently associated with a higher risk of HIV disease progression, AIDS events, and all-cause mortality.

Allavena, Delpierre et al. (2012), Journal of Antimicrobial Chemotherapy	France	2994 HIV-positive patients.	55.6% had 25(OH)D <30 ng/mL. 31.1% had 25(OH)D <10 ng/mL.	No relationship was found in duration since HIV diagnosis and vitamin D deficiency.

Meyzer, Frange et al. (2013), Pediatr Infect Dis J	France	113 HIV-infected children versus 54 healthy controls.	70% versus 45% had 25(OH)D <30 ng/mL. 25% versus 55% had 25(OH)D <10 ng/mL.	Dark phototype was the only independent risk factor for vitamin D deficiency in HIV-infected children.

Theodorou et al. (2014) [29], Clinical Nutrition	Belgium	2044 HIV-infected subjects.	89.2% had 25(OH)D <30 ng/mL. 32.4% had 25(OH)D <10 ng/mL.	The authors also found a positive association between AIDS diagnosis and vitamin D deficiency; in particular, it was associated with cART modalities and duration.

Van Den Bout-Van Den Beukel et al. (2008) [52], AIDS Research and Human Retroviruses	Netherlands	252 HIV-positive patients.	28.96% had 25(OH)D <35 nmol/L from April to September and <25 nmol/L from October to March.	Female sex, younger age, dark skin, and NNRTI treatment were significant risk factors in univariate analysis, although in multivariate analyses skin pigmentation remained the only independent risk factor.

Bang, Shakar et al. (2010), Scand J Infect Dis	Denmark	115 HIV-positive patients.	20.0% had 25(OH)D <25 nmol/L. 4.0% had 25(OH)D <12.5 nmol/L.	Vitamin D level was not associated with age, with HIV infection, highly active antiretroviral therapy (HAART) or CD4 count.

Welz, Childs et al. (2010) AIDS	UK	1077 HIV-positive patients	91% 25(OH)D <30 ng/mL 33% 25(OH)D <10 ng/mL	Black ethnicity, sampling in winter, CD4 cell count lower than 200 cells/microl, and exposure to combination antiretroviral therapy were associated with severe vitamin D deficiency.

Gedela et al. (2014) [18], International Journal of STD & AIDS	UK	253 HAART-naive subjects.	58.5% had 25(OH)D <30 ng/mL. 12.6% had 25(OH)D <10 ng/mL.	25(OH)D deficiency was common among antiretroviral treatment-naive patients, with those of nonwhite ethnicity at highest risk; no association was found with CD4 count, HIV viral load, and HIV clinical staging.

Mueller, Fux et al. (2010), AIDS	Swiss	211 HAART-naive subjects.	42% had 25(OH)D <30 ng/mL in spring. 14% had 25(OH)D <30 ng/mL in fall.	Vitamin D status significantly changed in HIV-positive patients according to seasons, intravenous drugs use, and longer HIV diagnosis but remained unchanged regardless of combined cART exposure.

Haug, Aukrust et al. (1998), Journal of Clinical Endocrinology and Metabolism	Norway	54 HIV-positive patients.	54% had 1,25(OH)2D <95 pmol/L and 62% of them had undetectable levels.	HIV-patients had low 1,25(OH)2D levels, whereas they had normal serum levels of 25(OH)D and vitamin D-binding protein. Moreover, they had modestly depressed serum calcium and PTH levels. No correlations were found between these parameters and serum levels of 1,25(OH)2D. Patients with undetectable 1,25(OH)2D were characterized by advanced clinical HIV infection, low CD4+ lymphocyte counts, and high serum levels of TNF-alpha. Inadequate 1alpha-hydroxylation of 25(OH)D could be the cause of 1,25(OH)2D deficiency, possibly induced by an inhibitory effect of TNF-alpha.

Vescini, Cozzi-Lepri et al. (2011), Journal of Acquired Immune Deficiency Syndromes	Italy	810 HIV-positive patients.	47% had 25(OH)D <30 nmol/L. 3% had 25(OH)D <10 nmol/L.	Authors highlighted a correlation between 25(OH)D insufficiency and risk of cardiovascular events, diabetes mellitus, and renal disease over a median 6.5-year follow-up. 25(OH)D levels below 30 nmol/L seemed to predict faster HIV progression.

Pinzone, Di Rosa et al. (2013), Eur. Rev. Med. Pharmacol. Sci.	Italy	91 HIV-positive patients.	57% patients had 25(OH)D <30 ng/mL. 31% patients had 25(OH)D <10 ng/mL.	Vitamin D deficiency was common in HIV-infected patients. Chronic inflammation, including residual viral replication, may contribute to 25(OH)D reduction modulating vitamin D metabolism and catabolism.

Cervero, Agud et al. (2012), AIDS Research and Human Retroviruses	Spain	352 HIV-positive patients.	71.6% had 25(OH)D <30 ng/mL. 44.0% had 25(OH)D <20 ng/mL.	Higher body mass index, black race, lower seasonal sunlight exposure, men who have sex with men and heterosexual transmission categories, efavirenz exposure, and lack of HIV viral suppression were independently associated with 25(OH)D deficiency/insufficiency.

Lerma, Molas et al. (2012), ISRN AIDS	Spain	566 HIV-positive patients.	71.2% had 25(OH)D <30 ng/mL; 39.6% had 25(OH)D <20 ng/mL.	Nonwhite race and psychiatric comorbidity were predictors of vitamin D deficiency.

Teichmann et al. (2000) [56], Journal of Infection	Germany	54 HIV-positive females prior to HAART versus 50 healthy women.	1,25(OH)2D levels in HIV-positive women, 19.4 ± 7.2; 1,25(OH)2D levels in healthy women, 47.3 ± 9.1; 25(OH)D levels in HIV-positive women, 37.3 ± 7.9; 25(OH)D levels in healthy women, 61.5 ± 8.4.	Lumbar osteoporosis was found in 7 patients (14%) versus 0 controls; lumbar osteopenia was diagnosed in 31 (62%) patients and 2 (4%) controls. There was significant correlation between the CD4 counts and 1,25(OH)2D levels. Neither the CD4 counts nor the duration of disease correlated with BMD

Etminani-Esfahani, Khalili et al. (2012), Current HIV Research	Iran	98 HIV-positive patients.	86.7% had 25(OH)D <35 nmol/L.	Female sex, unemployment, and human hepatitis C coinfection were related to the severe serum vitamin D deficiency.

Bajaj, Misra et al. (2012), Indian Journal of Endocrinology and Metabolism	India	45 HIV-positive patients. 45 healthy controls.	93.33% patients had 25(OH)D <30 ng/mL. 73.33% patients had 25(OH)D <30 ng/mL.	51.11% patients had dyslipidemia compared to 15.55% of controls. A positive association was proved between CD4 levels and 25(OH)D. No significant difference was seen in carotid intima-media thickness in cases and controls.

Conrado, Miranda-Filho Dde et al. (2011), Journal of the International Association of Providers of AIDS Care (Chic)	Brazil	214 HIV-positive female patients on cART.	40.65% patients had 25(OH)D <30 ng/mL.	Multivariate analysis proved that hypercholesterolemia and cART ≥3 years were positively associated with 25(OH)D deficiency, whereas there was an inverse statistically significant correlation with total cholesterol.

Wiboonchutikul, Sungkanuparph et al. (2012), Journal of the International Association of Providers of AIDS Care (Chic)	Thailand	178 HIV-positive patients.	44.9% had 25(OH)D <30 ng/mL and 26.8% had 25(OH)D <20 ng/mL.	Efavirenz intake was significantly associated with low vitamin D status. The mean 25(OH)D levels in patients receiving and not receiving EFV were, respectively, 22.9 and 28.6 ng/mL.

Conesa-Botella, Goovaerts et al. (2012), International Journal of Tuberculosis and Lung Disease	Uganda	92 HIV-positive TB-positive patients (G1). 20 only HIV-positive TB-negative patients (G2). HIV-negative TB-positive. 23 HIV-negative TB-negative patients (G4).	41% patients of G1 had 25(OH)D <75 nmol/L. 35% patients of G2 had 25(OH)D <75 nmol/L. 37% patients of G3 had 25(OH)D <75 nmol/L. 65% patients of G4 had 25(OH)D <75 nmol/L.	The authors reported that the prevalence of optimal vitamin D status was relatively high in HIV-infected patients with and without TB living in Uganda near the equator.

Mastala, Nyangulu et al. (2013), PLoS One	Malawi	69 HIV-positive of 157 TB negative patients.	23.1% of HIV-positive patients had 25(OH)D <50 nmol/L.	25(OH)D deficiency seemed more common in TB patients than non-TB patients. No significant correlation was found with HIV-status.

Rwebembera, Sudfeld et al. (2013), J Trop Pediatr	Tanzania	191 HIV-exposed uninfected infants.	48.7% had 25(OH)D <30 ng/mL. 34.6% had 25(OH)D <20 ng/mL.	25(OH)D deficiency was associated with sampling during the rainy season and infant wasting, whereas infant breastfeeding, maternal CD4 T-cell count, maternal wasting status, and maternal receipt of cART were not associated.

Havers et al. (2014) [24], The Journal of Infectious Diseases	US and 8 resource-limited countries	411 patients from PEARLS trial.	49% had 25(OH)D <32 ng/mL.	25(OH)D deficiency ranged from 27% in Brazil to 78% in Thailand. It was associated with high body mass index, winter/spring season, country-race group, and lower viral load. In addition, baseline low 25(OH)D was associated with increased risk of HIV progression, death, and virologic failure after cART.

1,25(OH)(2)D: 1,25-dihydroxyvitamin D; 25(OH)D: 25-hydroxyvitamin D; AIDS: acquired immune deficiency syndrome; cART: combined antiretroviral therapy; BMD: bone mass density; HAART: highly active antiretroviral therapy; NNRTI: nonnucleoside reverse-transcriptase inhibitor; PI: protease inhibitor; PTH: parathyroid hormone; US: United States; TB: tuberculosis; TNF-alpha: tumor necrosis factor-alpha; UK: United Kingdom.

**Table 2:** Association between HAART use and low 25(OH)D levels.

Authors (year), journal	Nation	Patients	Results	Comments
Poowuttikul, Thomas et al. (2014), Journal of the International Association of Providers of AIDS Care	US	160 HIV-infected youth (45 no ART; 67 cART with tenofovir or EFV; 48 other cART).	25(OH)D in tenofovir/EFV group: 20.3 ± 18.1 ng/mL. 25(OH)D in other cART group 21.2 ± 16.8 ng/mL. 25(OH)D in no ART group 14.6 ± 7.3 ng/mL.	Severe vitamin D deficiency (25(OH)D ≤10 ng/mL) was related to lower CD4 counts and CD4% but not to HIV plasma RNA. EFV or tenofovir therapy did not have different effects on vitamin D levels compared to other antiretroviral medications.

Viard et al. (2011) [21], AIDS	31 European countries, Israel, and Argentina	1985 HIV-positive among EuroSIDA study group (180 naive, 155 ART, and 1650 cART).	36.6% naive had 25(OH)D <12 ng/mL. 39.3% ART had 25(OH)D <12 ng/mL. 35.5% cART had 25(OH)D <12 ng/mL. 38.8% naive had 25(OH)D <20 ng/mL. 32.2% ART had 25(OH)D <20 ng/mL. 30.4% cART had 25(OH)D <20 ng/mL.	25(OH)D deficiency was frequent in HIV-infected persons (83% on combined antiretroviral therapy) and was independently associated with a higher risk of mortality and AIDS events. Patients receiving a PI-based antiretroviral regimen were at low risk of hypovitaminosis D, whereas no significant association was found with EFV or tenofovir use.

Allavena, Delpierre et al. (2012), Journal of Antimicrobial Chemotherapy	France	2994 HIV-positive patients (334 cART naive versus 2660 exposed).	79.3% had 25(OH)D <30 ng/mL among ART naive. 67.6% had 25(OH)D <30 ng/mL among cART exposed.	In multivariate analysis cART, treatment was associated with vitamin D deficiency (aOR 2.61), together with current smoking, estimated glomerular filtration rate ≥90 mL/min/1.73 m^2^, vitamin D measurement not performed in summer, and CD4 <350 cells/mm^3^.

Theodorou et al. (2014) [29], Clinical Nutrition	Belgium	2044 HIV-infected subjects.	1500 (73.4%) patients under HAART. 1362 (74.7%) patients under HAART had 25(OH)D <30 ng/mL.	25(OH)D levels varied according the different combinations of cART (*P* < 0.0001). Median 25(OH)D levels in patients treated with 2 NRTI + 1 NNRTI and patients 2 NRTI + 1 PI were 12.5 ng/mL versus 14.3 ng/mL, respectively, (*P* = 0.0001).

Welz, Childs et al. (2010), AIDS	UK	755/1077 HIV-positive, patients under cART.	52.1% patients under cART had 25(OH)D <10 ng/mL.	EFV treatment was significantly associated with severe 25(OH)D reduction (OR: 2.0). Tenofovir (OR: 3.5) and EFV use OR: 1.6), but not severe 25(OH)D deficiency (OR: 1.1), was associated with increased bone turnover.

Cervero, Agud et al. (2012), AIDS Research and Human Retroviruses	Spain	352HIV-positive patients (37 cART naive versus 315 cART exposed).	95.2% had 25(OH)D <30 ng/mL among cART naive. 68.4% had 25(OH)D <30 ng/mL among cART exposed.	EFV exposure was associated with 25(OH)D deficiency (*P* = 0.018). Patients receiving PIs (*P* = 0.014) or NNRTI (*P* = 0.025) had higher odds of increased PTH levels; this was significant only in 25(OH)D deficient patients (*P* = 0.004).

Van Den Bout-Van Den Beukel et al. (2008) [52], AIDS Research and Human Retroviruses	Netherlands	252 HIV-positive patients.	25(OH)D levels in white NNRTI-treated patients: 54.5 (27.9–73.8) nmol/L; 25(OH)D levels in white PI-treated patients 77.3 (46.6–100.0) nmol/L. 25(OH)D levels in black NNRTI-treated patients: 22.0 (14.7–38.4) nmol/L. 25(OH)D levels in black PI-treated patients 29.0 (20.4–5) nmol/L.	Female sex, younger age, dark skin, and NNRTI treatment were significant risk factors in univariate analysis, although in multivariate analyses skin pigmentation remained the only independent risk factor.

Fox, Peters et al. (2011), AIDS Research and Human Retroviruses	12 countries in Europe	256 European patients taking EFV + 2NNRTI or PI + 2NNRTI.	25(OH)D on PI + 2NNRTI 41.6 (38.6, 44.5) nmol/L. 25(OH)D on EFV + 2NNRTI 35.0 (31.0, 39.1) nmol/L.	Lower baseline vitamin D levels were associated with EFV (*P* = 0.0062) and zidovudine (*P* = 0.015) use. The increase in 25(OH)D values in about 27% of patients who discontinued EFV (*P* = 0.007) was relevant.

Brown and McComsey (2010) [32], Antivirus Therapy	US	51 HIV patients under EFV-containing treatment. 36 HIV patients under non-EFV-containing treatment.	Median 25(OH)D level before cART 52.7 nmol/L 25(OH)D reduction in EFV-treated versus non-EFV-treated patients: −12.7 ± 3.7 nmol/L.	A significant decline in 25(OH)D serum levels after the initiation of an EFV-based regimen, compared to a non-EFV-based regimen (*P* < 0.001) in HAART patients was found. In addition, subjects receiving EFV had a 1.8-fold increased probability of developing vitamin D deficiency, compared to those starting PIs.

Conesa-Botella, Florence et al. (2010), AIDS Research and Therapy	Belgium	89 HIV-positive patients before and after 12-month HAART.	43.7% had 25(OH)D <20 ng/mL before HAART and 47.1% before 12-month HAART. 70.1% had 25(OH)D <20 ng/mL before HAART and 81.6% before 12-month HAART.	A 3-fold increased risk of 25(OH)D levels below 20 ng/mL was described in subjects receiving NNRTIs (*P* = 0.02) after 12 months of HAART.

Schwartz, Moore et al. (2014), Journal of the International Association of Providers of AIDS Care	US	507 HIV-negative subjects. 358 HIV-positive patients cART naive. 893 HIV-positive patients under cART.	72% HIV-negative subjects had 25(OH)D <20 ng/mL. 18% HIV-negative subjects had 25(OH)D <30 ng/mL. 70% HIV-positive patients ART naive had 25(OH)D <20 ng/mL. 20% HIV-positive patients ART naive had 25(OH)D <30 ng/mL. 57% HIV-positive patients under cART had 25(OH)D <20 ng/mL. 24% HIV-positive patients under cART had 25(OH)D <30 ng/mL.	EFV use in cART significantly reduced the 25(OH)D levels (15 versus 19 ng/mL; *P* < 0.001). Hypertriglyceridemia was present in HIV-infected under ART (13% versus 7% of HIV-infected cART and 5% of HIV-uninfected; *P* < 0.001), with a positive relationship between 25(OH)D levels and triglycerides (*P* < 0.01). No relationships could be found between 25(OH) and cholesterol. Vitamin D deficiency was not correlated to HIV status but influenced by HIV treatment.

Fux, Baumann et al. (2011), AIDS	Switzerland	262 HIV-positive patients starting HAART (EFV versus PIs).	40.6% under EFV had 25(OH)D <30 nmol/L after 1-year therapy and 25.0% under PIs had 25(OH)D <30 nmol/L after 1 year therapy.	EFV treatment was associated with lower 25(OH)D levels compared to PIs. CYP polymorphisms and black ethnicity may define patients in whom EFV treatment will cause clinically relevant 25(OH)D deficiency.

Pasquet, Viget et al. (2011), AIDS	France	352 HIV-positive patients under cART.	41.0% patients under cART had 25(OH)D <30 nmol/L.	Authors found an association between hypovitaminosis D and exposure to NNRTIs (*P* = 0.05) but not to EFV and NVP, probably because of a lack of statistical power of their analysis. However, considering the crude and adjusted coefficients for EFV and NVP in their regression models, the authors suggested a NNRTI class effect, rather than a specific EFV or NVP impact, on vitamin D levels.

Ryan, Dayaram et al. (2013), Current HIV Research	US	1368 naive HIV-positive patients (686 cART with RPV; 682 cART with EFV).	In EFV arm median 25(OH)D reduction after therapy was greater in older (–3.2 ng/mL) versus younger (–1.6 ng/mL). In RPV arm median 25(OH)D remained relatively unchanged for both older (0.8 ng/mL) and younger (–0.8 ng/mL).	Progression from insufficient (50–74 nmol/L) or deficient (25–49 nmol/L) at baseline to severely deficient (<25 nmol/L) 25(OH)D at week 48 after cART was 0% in older and 2% in younger under RPV, whereas it was 13% in older and 8% in younger under EFV.

Wohl et al. (2014) [30], Antivirus Therapy	US	690 naive HIV-positive patients (345 cART with RPV; 345 cART with EFV).	In EFV arm median 25(OH)D reduction after 48-week therapy was (–2.5 ng/mL). In RPV arm median 25(OH)D reduction after 48-week therapy was (–0.2 ng/mL).	Patients with severe 25(OH)D deficiency were 5% in both groups at baseline but were significantly higher in EFV group at 48 weeks (9% versus 5%, *P* = 0.032). In addition, the patients with 25(OH)D insufficiency/deficiency at baseline, the ones who received EFV, developed more frequently severe 25(OH)D deficiency (8% versus 2%, *P* = 0.0079).

Viani, Peralta et al. (2006), The Journal of Infectious Diseases	US and Puerto Rico	303 HIV-positive patients under cART (102 received vitamin D supplementation, the others placebo).	At baseline, 54% had 25(OH)D <20 ng/mL. 45% of treatment group had 25(OH)D <20 ng/mL. 93% of treatment group had sufficient 25(OH)D levels after 12 weeks of therapy.	Oral vitamin D supplementation (50,000 IU monthly) increased 25(OH)D serum concentration from a baseline of 21.9 (13.3) to 35.9 (19.1) ng/mL after 12 weeks (*P* < 0.001) with no change for placebo. Although use of the antiretroviral efavirenz was associated with lower baseline 25-OHD concentration, efavirenz did not diminish the response to vitamin D supplementation. No toxicity was revealed.

1,25(OH)(2)D: 1,25-dihydroxyvitamin D; 25(OH)D: 25-hydroxyvitamin D; ART: antiretroviral therapy; cART: combined antiretroviral therapy; EFV: efavirenz; HAART: highly active antiretroviral therapy; NNRTI: nonnucleoside reverse-transcriptase inhibitor; NRTI: nucleoside reverse-transcriptase inhibitor; PI: protease inhibitor; RPV: rilpivirine; US: United States.

**Table 3:** Carotid intima-media thickness (cIMT) and hypovitaminosis D in HIV patients.

Authors (year), journal	Nation	Patients	Results	Comments
Bajaj, Misra et al. (2012) Indian Indian Journal of Endocrinology and Metabolism	India	45 HIV-positive patients and 45 controls.	93.33% patients had 25(OH)D <30 ng/mL. 73.33% controls patients had 25(OH)D <30 ng/mL. cIMT 6 mm, 51.11% patients. cIMT 7 mm, 15.55% patients. cIMT 8 mm, 13.33% patients. cIMT >8 mm, 0% patients.	No significant difference in cIMT was proved between HIV-positive patients and controls (*P* = 1.00). A positive association was seen between CD4 levels and 25(OH)D.

Ross et al. (2011) [16], Antiviral Therapy	US	149 HIV-positive patients (56 with carotid IMT), 34 controls.	5% patients had 25(OH)D <25 nmol/L. 46% patients had 25(OH)D <50 nmol/L. Mean icIMT in HIV-patients: 0.70 (0.55–0.91). Mean ccIMT in HIV-patients: 0.65 (0.55–0.75).	Authors observed a 10.62 higher probability of having cIMT above the median value in HIV-infected adults with 25(OH)D values below 30 ng/mL (*P* = 0.01). Vitamin D status was associated with CD4+ T-cell restoration after antiretroviral therapy but not with the inflammatory and endothelial activation markers, soluble TNF-*α* receptor 1 (sTNFR-1), and soluble intercellular adhesion molecule-1 (sICAM-1), associated with atherosclerosis and CVD development in the general population.

Choi et al. (2011) [45], Clinical Infectious Diseases	US	139 HIV-positive patients.	52% had 25(OH)D <30 ng/mL. Mean cIMT in patients with 25(OH)D >30 ng/dL: 0.87 mm. Mean cIMT in patients with 25(OH)D <30 ng/dL: 1.0 mm. Mean cIMT in patients with 25(OH)D <15 ng/dL: 1.1 mm.	An association between vitamin D insufficiency and cIMT, even after adjusting for age, sex, tobacco use, hypertension, and elevated cholesterol, was proved. The authors found that mean cIMT was 0.13 mm greater in vitamin D insufficient subjects than in normal subjects.

Eckard et al. (2013) [12], The Pediatric Infectious Disease Journal	US	30 HIV-positive patients, 31 controls.	72% patients versus 87% controls had 25(OH)D <20 ng/mL. 21% patients versus 13% controls had 25(OH)D <30 ng/mL.	After adjusting for season, sex, and race, there was no difference in serum 25(OH)D between groups (*P* = 0.11). Serum 25(OH)D was not significantly correlated with cIMT (*P* = 0.34). In HIV-infected group, 25(OH)D was negatively correlated with HOMA-IR, HIV duration, and cumulative duration of ART, NRTI, and NNRTI duration.

Portilla et al. (2014) [46], Journal of the International AIDS Society	Spain	89 HIV-positive patients (75 on ART).	80.8% had 25(OH)D <75 nmol/L. Bilateral mean cIMT in 25(OH)D deficient 0.63 ± 0.08 versus not deficient 0.56 ± 0.06 (*P* = 0.09).	High prevalence of 25(OH)D (80.9%) was found. Authors found no association between 25(OH)D insufficiency, inflammatory, or endothelial dysfunction markers and cIMT, whereas this was found between cIMT and patient age, impaired fasting glucose, and PI therapy length.
				

25(OH)D: 25-hydroxyvitamin D; ART: antiretroviral therapy; ccIMT: common carotid intima-media thickness; cIMT: carotid intima-media thickness; US: United States; icIMT: internal carotid intima-media thickness; CVD: cardiovascular diseases; HOMA-IR: homeostasis model assessment of insulin resistance; NNRTI: nonnucleoside reverse-transcriptase inhibitor; NRTI: nucleoside reverse-transcriptase inhibitor.

**Table 4:** Brachial artery flow-mediated dilation, coronary artery calcium (or calcification) and hypovitaminosis D in HIV patients.

Authors (year), journal	Nation	Patients	Results	Comments
Lai et al. (2013) [47], Vascular Health and Risk Management	US	846 HIV-infected African-American participants.	28.1% had CAC.	Logistic regression analysis revealed the factors independently associated with CAC: age, male sex, family history of CAD, years of cocaine use, total cholesterol, high-density lipoprotein cholesterol, PI treatment length, and, finally, vitamin D deficiency.

SShikuma, Seto et al. (2012), AIDS Research and Human Retroviruses	US (Hawaii)	100 patients of the HIV-Cardiovascular Cohort Study	Median 25(OH)D: 27.9 ng/mL. CAC was present in 53%.	A significant correlation was observed between 25(OH)D levels and FMD (*P* = 0.01) but not with cIMT (*P* = 0.76). Lower 25(OH)D levels were at slightly higher risk of having CAC (*P* = 0.04); these lower 25(OH)D levels were not associated with higher CAC scores (*P* = 0.36).

Gepner, Ramamurthy et al. (2012), PLoS One	US	114 healthy postmenopausal women (54 treated with vitamin D supplementation and 57 with placebo).	Median pretreatment 25(OH)D 30.3 in treatment group, 32.3 in placebo group. FMD pretreatment 0.018 in treatment group, 0.016 in placebo group. FMD posttreatment 0.001 in treatment group, 0.001 in placebo group.	Authors proved no improvement in endothelial function, arterial stiffness (as measured by brachial artery FMD, carotid-femoral pulse wave velocity, and aortic augmentation index), or inflammation markers after vitamin D supplementation in the general population.

de Boer, Kestenbaum et al. (2009), Journal of the American Society of Nephrology	US	1370 HIV-negative patients (394 with and 976 without CKD).	53% had CAC at baseline (65% with CKD and 48% without CDK). 21% of subjects who do not have CAC at baseline developed it during 3-year follow-up.	Lower 25(OH)D concentration was associated with increased risk for CAC development; each 10 ng/mL 25(OH)D reduction there was a 23% increased risk (*P* = 0.049).

25(OH)D: 25-hydroxyvitamin D; CAC: coronary artery calcification; cIMT: carotid intima-media thickness; CKD: chronic kidney disease; FMD: artery flow-mediated dilation; PI: protease inhibitor; US: United States.
